# Application of Physiological Sperm Selection in Assisted Reproductive Technology (ART) for Patients With Diabetes Mellitus: A Case Report

**DOI:** 10.7759/cureus.53018

**Published:** 2024-01-26

**Authors:** Krushnali S Kadu, Akash More, Namrata Choudhary, Shilpa Dutta, Jarul Shrivastava, Gauri Gajabe

**Affiliations:** 1 Clinical Embryology, Datta Meghe Institute of Higher Education & Research, Wardha, IND

**Keywords:** macs, infertility, picsi, hyaluronic acid, sperm dna fragmentation

## Abstract

This case report revolves around a 35-year-old woman and her 39-year-old husband, who have been married for 10 years and were seeking treatment for their secondary infertility. The husband had been diagnosed with diabetes mellitus (DM) for the past seven years and had been on continued medication. Both partners underwent medical evaluations, and the husband was found to have normozoospermia. The wife had a normal ovarian reserve but was still facing difficulties in conception. Previous medical history showed that the husband’s DM had been treated with metformin, and the woman had a history of unsuccessful in vitro fertilization (IVF) cycles and one miscarriage upon investigation. Our initial treatment attempt had failed, and upon assessment of the semen, it revealed a significantly increased sperm DNA fragmentation index, leading us to consider physiological intracytoplasmic sperm injection (PICSI). The couple then opted for a rescheduled ovum pick-up with PICSI over traditional ICS. PICSI involves selecting mature sperm with hyaluronic acid affinity, aiming to avoid immature, morphologically defective spermatozoa during microinjection. The couple then followed up for treatments; the husband continued with diabetes treatment, and the woman was advised to take progesterone supplements to ensure optimum uterine thickness. The PICSI procedure was followed by a successful embryo transfer, which subsequently led to a positive clinical pregnancy. This report highlights the importance of utilizing advanced technologies like PICSI in infertility cases after considering factors such as sperm quality to enhance the chances of a successful clinical pregnancy.

## Introduction

Infertility has been defined as a condition in which a male or female is unable to conceive even after one year of regular, unprotected sexual intercourse [1]. This condition is unique in that it affects both males and females. A total of 40-50% of marriages are affected by male factor infertility. Given the high frequency of male factors in infertile heterosexual couples, an initial medical history and assessment of the male partner are suggested [2]. In Australia, between September and December 1988, at least 1,495 couples in Perth completed a survey as part of a study to determine the extent of infertility [3]. A recent study in India by Sarkar et al. says that around 8% of married women suffered from primary infertility and secondary infertility, and 5.8% of them were secondary infertile [4]. Also, the earlier studies demonstrate that the metabolism of glucose is a very important factor for sperm, and type 1 or type 2 diabetes can have harmful effects on the fertility of the male partner, especially on the quality of sperm cells, like motility, the integrity of sperm DNA, and the composition of semen [5]. There are many reasons for embryo transfer failure, including endometrial thickness, receptivity, embryo abnormalities, and inherited factors [6].

In situations of abnormal sperm DNA fragmentation (SDF), physiological intracytoplasmic sperm injection (PICSI) and magnetic-activated cell sorting (MACS) are beneficial sperm selection methods. However, MACS is more common in females under the age of 30, and PICSI is preferred in older females [7]. In conditions of unexplained infertility, methods for separating sperm with low DNA fragmentation can be utilized. The purpose of this study was to assess the efficacy of PICSI in patients with uncertain infertility [7]. Sperm quality has a significant role in controlling embryo growth and, consequently, the success of ICSI. One way of assessing sperm quality has been suggested: selecting morphologically normal sperm based on their capacity to attach to hyaluronic acid (HA). The purpose of this examination was to see if injecting HA-bound sperm improves outcomes in individuals undergoing ICSI for unexplained infertility with normal sperm parameters [8]. HA can potentially improve ICSI results by making it less difficult to identify and select spermatozoa with intact nuclei and minimal DNA damage. In addition, HA can be used to speed up the selection of spermatozoa with normal nuclei in the context of intracytoplasmic morphologically selected sperm injection [9]. Sperm quality is critical to regulating embryonic growth and the success of ICSI. One way of assessing sperm quality has been suggested: selecting suitable sperm based on its capacity to attach to HA [8]. A recent study has shown that using HA as a medium may have a greater beneficial effect on clinical pregnancy success [6].

PICSI works by choosing sperm based on the oocyte’s affinity for HA, leading to higher ICSI success rates [10]. This study has been conducted to highlight the effect of PICSI on the in vitro fertilization (IVF) journey of patients suffering from unexplained infertility, and it thereafter resulted in a positive clinical pregnancy.

## Case presentation

Patient information

After 10 years of marriage, a 35-year-old female came to our clinic with her 39-year-old husband, who was suffering from infertility. They had been trying to conceive for three years. They consulted a doctor after three years due to having a preconceived notion about IVF. The female patient was a housewife, and her husband worked as a farmer. They had been together for 10 years, and the husband had diabetes mellitus (DM) for the past seven years.

Medical/surgical history

The male had a medical history of DM. The female had a past medical history of two failed IVF cycles at different IVF centers. The female also had a medical history of one miscarriage via IVF. This was a case of secondary infertility. The woman had a BMI of 22, and her husband’s BMI was 25, both within the normal range. No infertility-related results were reported.

Investigation

The husband’s semen analysis reported normozoospermia, i.e., a normal sperm count of 35 mL, with decreased (29%) progressive motility. The husband’s hormonal examination found diabetes. Random blood sugar tests were done to assess the sugar level in the person; reports suggested that the husband had been diabetic for seven years, with SDF showing only 10% large halo, 20% small halo, and 70% degraded sperm.

For the female partner, a transvaginal ultrasound was performed to investigate her ovarian reserve and look for any structures of abnormality. The female partner has an anti-Müllerian hormone (AMH) level of 1.6 ng/dL. Follicle-stimulating hormone had a value of <6.9 mIU/mL, and luteinizing hormone had a value of 2.59 mIU/mL. Hysterosalpingography was performed to determine tubal patency, which is an examination for female infertility that determines whether the fallopian tubes are open or not. The result was found to be normal.

Diagnosis

This is a case of secondary infertility. The woman had an AMH level of 1.6 ng/dL, and her husband was normozoospermic.

Treatment

The couple visited an assisted reproductive technology clinic in a rural region. The male had a past medical history of DM. The patient had suffered from diabetes for seven years. The female also had a medical history of two IVF cycle failures and one miscarriage. The semen analysis of the male partner reported normal parameters. This denoted that the male partner had normozoospermia. The female partner also underwent rigorous clinical assessment, and her reports came out to be normal. Hence, it was inferred from the clinical reports that the couple had unexplained infertility.

This was an instance of secondary infertility, which might have been caused by diabetes. We started treatment for diabetes. The medicine suggested by the doctor was metformin, and a daily dose of 500 mg was advised after having his evening meal. After the treatment of diabetes, the patient was scheduled for ovum pick-up (OPU), started medication progesterone, and was given a human chorionic gonadotropin (hCG) trigger 36 hours before the procedure of OPU. In the first OPU cycle, 13 oocytes were successfully retrieved during this procedure. Two oocytes of the M2 phase were retrieved, while one oocyte remained in the germinal vesicle stage, representing a developmental phase prior to maturity. A semen sample from the male patient was processed, ICSI was performed using the processed sample, and the embryo was cultured until day 5. Average-quality embryos were observed, and since all other conditions were normal, on day 5, we took a chance and transferred the blastocyst-grade embryo. After 14 days of embryo transfer, a β-hCG test was performed to determine the result, but the result was negative.

In order to understand the cause of implantation failure, the patient was advised to undergo an SDF test. The reports suggested that 40% of sperm were fragmented. Hence, it was concluded that a high level of DNA fragmentation may have been the cause of implantation failure. We suggested that the possibility of fertilization due to PICSI may increase the chances of fertilization success. After the results of the examination, the couple agreed on the PICSI procedures.

**Figure 1:**
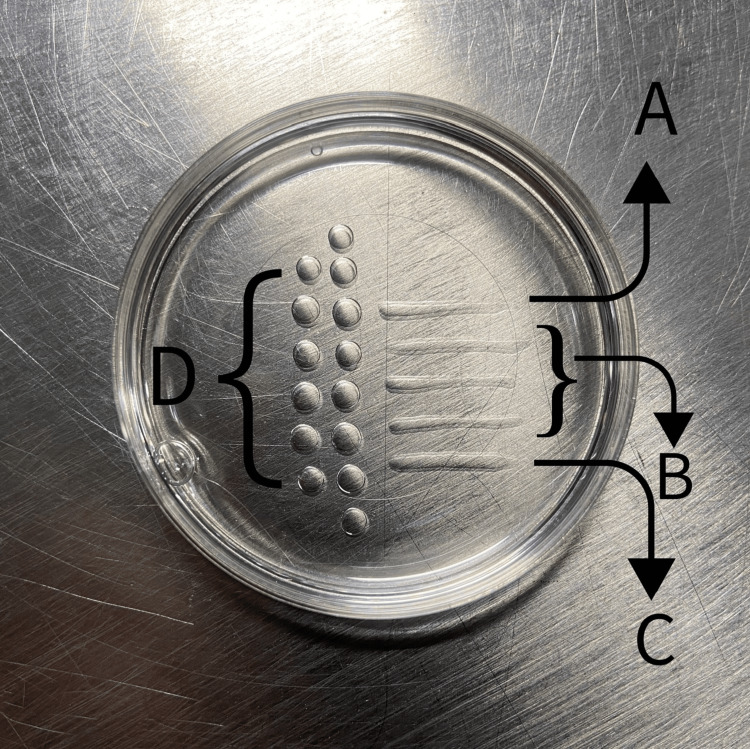
ICSI dish with (A and C) PVP line, (B) HA drops, and (D) 13 drops of buffer medium HA, hyaluronic acid; ICSI, intracytoplasmic sperm injection; PVP, polyvinylpyrrolidone

The patient was rescheduled for OPU, followed by the PICSI procedure (Figure 1 shows the PICSI dish) before ICSI. It was observed that the embryos formed were of 4AB grade, a significant improvement from the previous cycle. The embryos were transferred on day 5.

Follow-up

The patient was advised to take the necessary precautions for 14 days post-embryo transfer and to maintain a nutritious diet. Heavy lifting was restricted for the time period. Then, for confirmation of clinical pregnancy, the patient was suggested to undergo a β-hCG blood test two weeks post-embryo transfer. The report suggested that the value of β-hCG was 956 mIU/mL.

## Discussion

The PICSI method is based on the fact that mature sperm heads have a particular receptor that enables them to attach HA, the main component of the cumulus oophorus. Immature spermatozoa, on the other hand, lack the ability to interact with HA [11].

Sperm immaturity highlights that spermatogenesis side effects may lead to abnormalities in sperm. This indicates the need for addressing sperm maturity in reproductive techniques. The PICSI method is described as a way to prevent the selection of immature spermatozoa before microinjection. This suggests that PICSI enhances the selection process, improving the overall quality of the sperm chosen for fertilization. Our goal is to clearly demonstrate the success rate of the PICSI method. The focus is on comparing the outcomes of the PICSI method with routine ICSI procedures [12].

According to the present study, spermatozoa attached to HA exhibit a significant reduction in nuclear anomalies and DNA fragmentation relative to spermatozoa obtained from polyvinylpyrrolidone (PVP). The injection of these specific spermatozoa after ICSI helps to improve the quality of the embryo. As a result, HA may improve the overall result of ICSI by promoting the selection of spermatozoa with intact DNA and a normal nucleus [9]. SDF levels are high in men with defective sperm quality and normozoospermic couples who are infertile [13].

In a study that involved 123 males with unexplained male infertility, the DNA fragmentation index (DFI) was determined in 120 individuals. A total of 26% of those individuals had DFI levels above 20%, which is associated with reduced fertility. Two groups were compared: normal ICSI (n = 140) and ICSI with spermatozoa selected for their capacity to attach to HA, called PICSI (n = 109). There were significantly higher fertilization rates in the PICSI group, with greater than 50% initial HA binding. The study suggests that using PICSI, where spermatozoa are selected based on their ability to attach to HA, resulted in higher fertilization rates, implantation rates, and clinical pregnancy rates compared to traditional ICSI in males with unexplained male infertility [14,15].

It appears that, even though the study was not specifically designed to examine miscarriage rates, the researchers observed a lower proportion of couples with clinical pregnancies experiencing miscarriage in the PICSI (ICSI with sperm selected based on attachment to HA) group compared to the standard ICSI group [16].

Statistical research shows positive outcomes with the use of the PICSI method. According to the study, individuals who used the PICSI method had significantly higher chances of becoming pregnant (4.62 times higher, according to the statistical research). While abortion rates were lower in the PICSI group, the study also shows successful clinical pregnancies with newborns alive and healthy, showing no abnormalities [17].

We utilized a Petri plate as a sperm selection device, incorporating three powdered HA drops. These HA drops were rehydrated using a culture medium before being employed in the PICSI dishes. After placing a single drop of semen at the edge of each HA drop for sperm selection, we observed that, despite demonstrating intense tail movement, mature HA-bound spermatozoa could be identified by their complete lack of progressive motion. PVP manipulates the good sperm. After a 15-minute incubation at 37°C, HA-bound sperm can be retrieved using a pipette and employed for ICSI [18]. Hence, PICSI can be an alternate line of treatment for people suffering from unexplained infertility.

## Conclusions

The PICSI procedure was performed on a couple suffering from unexplained infertility, and subsequently, it resulted in a positive clinical pregnancy. This serves as a testament to the fact that advanced semen techniques like PICSI can serve as an alternate procedure for couples suffering from unexplained infertility. This case has been conducted on a single patient, which is a limitation of the study. Hence, it is further recommended to perform additional studies, like randomized controlled trials, to validate the results of the study and thus establish it as a standard procedure for couples in these categories.
